# Sexual cues alter working memory performance and brain processing in men with compulsive sexual behavior

**DOI:** 10.1016/j.nicl.2020.102308

**Published:** 2020-06-10

**Authors:** C. Sinke, J. Engel, M. Veit, U. Hartmann, T. Hillemacher, J. Kneer, T.H.C. Kruger

**Affiliations:** a Hannover Medical School, Division of Clinical Psychology & Sexual Medicine, Department of Psychiatry, Social Psychiatry and Psychotherapy, Hannover, Germany; b Department for Psychiatry and Psychotherapy, Paracelsus Medical University Nuremberg, Prof. Ernst-Nathan-Str. 1, 90419 Nürnberg, Germany; c Hannover Medical School, Department of Psychiatry, Social Psychiatry and Psychotherapy, Hannover, Germany

**Keywords:** Compulsive sexual behavior, fMRT, Working memory, distraction

## Abstract

Pornography has been repeatedly at the centre of public attention and has been controversially discussed for a long time. However, little is known about the connection between pornographic stimuli and individual (neuronal) processing of attention and memory. Here, the impact and neural underpinnings of pornographic pictures on working memory processes in a sample of subjects with compulsive sexual behaviour was investigated. Therefore, whilst using functional magnetic resonance imaging (fMRI), a letter n-back task with neutral or pornographic pictures in the background was employed in 38 patients and 31 healthy controls. On the behavioural level, patients were slowed down by pornographic material depending on their pornography consumption in the last week, which was reflected by a higher activation in the lingual gyrus. In addition, the lingual gyrus showed a higher functional connectivity to the insula during processing of pornographic stimuli in the patient group. In contrast, healthy subjects showed faster responses when confronted with pornographic pictures only with high cognitive load. Also, patients showed a better memory for pornographic pictures in a surprise recognition task compared to controls, speaking for a higher relevance of pornographic material in the patient group. These findings are in line with the incentive salience theory of addiction, especially the higher functional connectivity to the salience network with the insula as a key hub and the higher lingual activity during processing of pornographic pictures depending on recent pornography consumption.

## Highlights

• Pornographic pictures affect working memory performance in an n-back task.
• Patients with compulsive sexual behaviour show slowed reaction times when presented with pornographic distractors.
• Decrease of performance is related to pornography consumption in the last week.
• Activity in lingual gyrus is associated with poorer performance.

## 1 Introduction

Pornography has been repeatedly at the centre of public attention and has been controversially discussed for a long time. Arguments range from the expression of sexual freedom as social progress to the cause of sexualized violence with disastrous effects. However, little is known about the connection between pornographic stimuli and individual (neuronal) processing of attention and memory. Through the easy accessibility, affordability and anonymity the internet offers nowadays, pornography consumption is constantly rising (Cooper, 1998, Lewczuk et al., 2019). However, excessive usage of pornography can be an indicator of compulsive sexual behaviour (CSB). CSB disorder is characterized by a persistent pattern of failure to control intense, repetitive sexual impulses or urges resulting in repetitive sexual behaviour and psychological strain (World Health Organization, 2018). Based on representative surveys, it is assumed that 3–7% of women and 10.3% − 11% of men are affected (Dickenson et al., 2018, Grubbs et al., 2019). However, it is not only characterized by excessive online pornography consumption but also can be shown through ‘real life’ behaviour, such as risky casual sexual relations or anonymous sex. The aetiology is currently unclear and CSB is often discussed in relation to addictions (Kraus et al., 2016), especially as neuroimaging studies have shown an involvement of the reward circuit in CSB, in particular concerning the ventral striatum (Brand et al., 2016, Gola and Draps, 2018, Gola et al., 2017, Voon et al., 2014). In addition, pornography consumption related differences in the striatum have also been observed in healthy subjects (Kühn and Gallinat, 2014). The higher striatal activity in CSB is most consistent with the incentive salience theory (IST) (Robinson and Berridge, 1993, Robinson and Berridge, 2008, Robinson et al., 2016), which differentiates between ‘wanting’ (e.g., craving) and ‘liking’ (e.g., pleasurable effects) in motivated behaviour. It proposes that the dopaminergic system renders certain stimuli associated with the motivated behaviour more salient (‘incentive salience’). A sensitization of the incentive increases salience through activation of the reward system, which can subsequently lead to addiction. Theoretically, the role of salience is to guide attention in a behaviourally relevant goal directed manner (Parr and Friston, 2017, Parr and Friston, 2019). Thus, salient stimuli should capture attention (Kerzel and Schönhammer, 2013). The observation that sexual stimuli are attracting attention could be demonstrated using a dot-probe task with sexual stimuli and a line orientation task (Kagerer et al., 2014). Also, using the dot-probe task, it could be shown that subjects excessively using online sexually explicit material had a greater attentional bias toward sexually explicit material (Mechelmans et al., 2014), leading to faster reaction times. However, for the dot-probe task, mixed data exist, as Prause et al. (2008) found faster (and not slower) reaction times towards sexual stimuli, but other tasks also indicate an attentional bias towards sexual stimuli. Using a visual probe task, attentional bias towards pornographic stimuli could be shown in healthy subjects (Pekal et al., 2018). Furthermore, an implicit positive association towards sexually explicit material in healthy subjects could be revealed using an approach-avoidance task (Sklenarik et al., 2019, Stark et al., 2017). In addition, attentional bias towards sexual reward was shown in CSB (Banca et al., 2016). Moreover, in a study with healthy male participants, it could be shown that working memory performance for pornographic material was impaired (Laier et al., 2013), but whether pornographic material draws attention away from working memory processes is not well investigated. On a neural level, it could be shown that the prolonged reaction time in a picture categorization task and a line orientation task on pornographic stimuli lead to prolonged reaction times and higher activation in the caudate nucleus, putamen, thalamus, ACC, and OFC, which was interpreted as an involvement of the reward system (Strahler et al., 2018).

Thus, we aim to investigate the interference of pornographic material with working memory processes by using functional magnetic resonance imaging (fMRI) during an n-back letter task with distracting pornographic and non-pornographic pictures in the background. We hypothesize that the more salient pornographic material draws attention away from the task, the more errors and/or prolonged reaction times will occur, as Fried and Johanson (2008) provided evidence to suggest that sexual content can be a distraction that interferes with the processing of the product information. In addition, we want to know whether individuals displaying excessive sexual behaviour are more prone to its distracting effect. This could be an indicator that pornographic material is a more salient stimulus for these subjects and would be in line with the IST as, according to the theory, addiction-related material should be more salient (Robinson et al., 2016). Therefore, we compare male subjects with CSB to healthy controls. Due to their preoccupation with sexuality (Kraus et al., 2016), subjects with excessive sexual behaviour should be more distracted by pornographic material and thus should perform worse/slower during the presentation of sexual stimuli. On the neuronal level, the distracting effect should be represented by differences in the frontoparietal attention network of these subjects compared to healthy controls.

## 2 Methods

### 2.1 Subjects

The described sample is a subsample of the SEX@BRAIN study, including all subjects who participated in the fMRI experiments. A detailed description of the recruitment and the overall sample can be found in Engel et al. (2019). Recruitment started with a press release, to which 539 men responded. Of these respondents, 201 could be reached by telephone for a pre-screening of Kafka’s proposed criteria (Kafka, 2010). If distress was predominantly caused by moral incongruence or violation of strict religious norms, subjects were not considered for participation. (see for example Lewczuk et al., 2020 for a discussion). In all, 73 of the screened subjects met at least three of these criteria. In the further process, 50 of the screened subjects decided to participate in the study. Three subjects were excluded post-hoc, as they did not reach the cut-off score of 53 on the Hypersexual Behaviour Inventory 19 (Reid et al., 2011). Control subjects were recruited using advertisements on the intranet of the Hannover Medical School. A total of 85 men responded, while 29 men did not respond to mail or phone. From the remaining 56 men, 38 men were included in the study. Participants were excluded due to intellectual disability (as measured by the Wechsler Adult Intelligent Scale-IV) (Wechsler, 2013), a psychotic disorder or acute psychotic episode (assessed with the Structured Clinical Interview for DSM-IV Axis 1 disorders (SCID-I)) (Wittchen et al., 1997), severe head injury, homosexual orientation on the Kinsey scale (Kinsey et al., 1948), and paedophilic sexual preference (assessed in a semi-structured interview). Behavioural and fMRI data were acquired in 81 heterosexual male subjects. We only screened for men with CSB, as these men seek help in consultation hours far more often and are better accessible. Subjects with homosexual orientation were excluded, as the explicit pornographic material shows male–female sexual interaction. Of the 50 included patients, five were not eligible for the MRI investigation due to MRI exclusion criteria and one subject due to medication affecting his sexual drive (salvacyl). Thus, 44 men were included as patients with hypersexual behaviour participated in the MRI experiment. The healthy control group comprised 37 subjects, whilst one could not participate in the MRI due to previously unknown claustrophobia. For the final analysis, six subjects had to be excluded due to excessive head movement (three per group with head movement > 2 mm), one patient due to a head injury, one control due to recent head trauma, one control participant due to a high HBI (but inconspicuous impression) based on the interview, one patient due to a low Hypersexual Behaviour Inventory (HBI) score (≤53) (but conspicuous impressions) based on the interview, one control subject due to a homosexual orientation and one patient due to incomplete data. Thus, MRI data of 38 patients and 31 controls were analysed. The study was conducted in accordance with the Declaration of Helsinki and was approved by the local ethics committee. Subjects gave written informed consent to participate, were free to withdraw from the study at any time and received reimbursement for their participation.

### 2.2 Psychological questionnaires

In order to access hypersexual behaviour, the HBI (Reid et al., 2011) and the revised version of the Sexual Addiction Screening Test (SAST-R) (Carnes et al., 2010) were used and analysed according to the manual. For HBI, a cut-off value of 53 was applied, while for the SAST-R, a cut-off value of 6 for the core items (1–20) was used. Also, a semi-structured interview was conducted accessing participants’ sexual characteristics, as well as the SIS/SES questionnaire (Janssen et al., 2002) to assess the trait sexual excitation/inhibition. For details, see Engel et al. (2019).

### 2.3 fMRI data acquisition

MRI data was acquired on a Siemens 3 T Skyra running Syngo VE11 using a standard 64 channel head coil. A total of 84 axial slices (resolution 2 × 2 × 2 mm) per volume were acquired in ascending order using a gradient simultaneous multislice EPI T2* sensitive sequences with the following parameters: repetition time (TR) = 1.55 s, echo time (TE) = 32 ms, flip angle = 90°, field of view = 256 × 256 mm and acceleration factor = 4. Prior to functional scans, an individual high resolution anatomical image was acquired for each participant using a T1 weighted magnetization prepared rapid acquisition gradient echo sequence (resolution 0.9 × 0.9 × 0.9 mm, TR = 2.3 s, TE = 3 ms, flip angle = 9° and field of view = 255 × 270 mm).

### 2.4 Experimental paradigm

This study was part of a series of experiments investigating subjects with hypersexual behaviour (Sex@Brain-Study). All subjects were asked to restrain from sexual activities 24 h prior to their participation. Here, we were interested in the distracting effect of explicit sexual material on working memory processes. Therefore, an n-back letter task was employed with distracting sexual and non-sexual pictures in the background. During this experiment subjects were confronted with explicit pornographic material for the first time in the whole study. The experiment was comprised of three factors: the between groups factor SEXUAL BEHAVIOUR (control/patient) as well as the within subject factors DIFFICULTY (1-back/2-back) and EXPLICITNESS (pictures showing couples jogging/couples during sexual intercourse). Before the task, subjects were allowed to practice the 1-back and 2-back version of the task without interfering pictures. One hour after the fMRI measurement, an unannounced recognition task was conducted to test whether memory retrieval of the background stimuli differed between patients and controls.

### 2.5 fMRI experiment

The fMRI experiment consisted of 24 blocks, six of each condition (1-back with explicit background pictures, 2-back with explicit background pictures, 1-back with neutral background pictures and 2-back with neutral background pictures), presented in randomized order with the restriction that no more than two blocks of the same condition were presented in a row. They all started with a presentation of the task instruction (1-back or 2-back) for 6 s. Then, each block had a duration of 20 s, where 10 letters (A–Z without mutated vowels, font size 80, font type: Arial and font colour: white) were shown with a task-irrelevant picture in the background. Each letter and background picture was visible for 1 s, followed by a fixation cross presented for 1 s. Within each block, three target letters were included in a random order. They all ended with an inter-block interval of 4–8 s (mean 6 s), where again a fixation cross was presented. Subjects were instructed to react to the target letter by pressing the right index finger on the response device.

### 2.6 Unannounced recognition task

One hour after the fMRI experiment, subjects participated in an unannounced recognition task that was performed outside the scanner. Here, the 80 pictures used in the experiment and 80 previously unknown pictures were presented, and subjects had to indicate their memory confidence on a 6-point rating scale (surely known, probably known, unsure known, unsure new, probably new and surely new). Each trial started with a fixation cross presented for 1 s. Then, the picture was presented for 2 s, followed by the confidence scale, which was presented until subjects had made their decision. This, in turn, triggered the next trial. Recognition accuracy was considered as the dependent variable.

### 2.7 Stimuli

The presentation of the stimuli and recording of the behavioural data were managed by using Presentation® software (Presentation 16.3, Neurobehavioral Systems Inc.,

Berkeley, CA, USA; www.neurobs.com) and was shown on a 32″ monitor from NordicNeuroLab (NNL) (Bergen, Norway; www.nordicneurolab.com), which was placed in front of the patient and visible via a mirror. Responses were collected with response grips from NNL.

### 2.8 Visual stimuli

The visual stimuli of the n-back task consisted of capital letters of the alphabet (A–Z). For background pictures, 20 pictures depicting heterosexual intercourse, 20 pictures depicting oral stimulation, 20 pictures depicting a couple taking a walk and 20 pictures depicting a couple jogging were used. The pictures were distributed equally on the different conditions. Thus, 10 intercourse pictures and 10 oral stimulation pictures were presented in the 1-back condition, while the other 20 pictures were used as background in the 2-back condition. The same held for the neutral condition. Each stimulus was presented three times for 2 s during the whole experiment.

### 2.9 fMRI image processing

DICOM images were converted to NIFTI format using dcm2nii. After removing the first five scans to compensate for T1 saturation effects, functional scans were then realigned. Afterwards, the mean echo planar image was co-registered to the individual T1 images. Structural and functional images were normalized to MNI space with a voxel size of 2 × 2 × 2 mm and smoothed with a 4 × 4 × 4 mm FWHM Gaussian kernel using SPM12.

### 2.10 Analysis of behavioural data

Behavioural data was automatically recorded by Presentation® and analysed using SPSS© (IBM Inc.). Statistical analyses were performed using two-tailed testing, and a p-value < 0.05 was considered statistically significant. All numbers, except reaction times, were indicated as the mean value ± standard deviation. For reaction times, the median ± standard deviation were analysed. Normal distribution was examined using the Kolmogorov-Smirnov test. As all dependent variables were normally distributed, parametric testing was used throughout. Correlations between functional and behavioural data were evaluated using Pearson’s correlation coefficient. Accuracy in the n-back and recognition task was transformed to the percentage of correct answers and arc-sine transformed in order to assure normal distribution.

### 2.11 fMRI analysis

Data analysis was performed using the General Linear Model (GLM). On the subject level, the model contained four regressors of interest modelling, the four experimental conditions (1-back with pornographic pictures (easy explicit), 2-back with pornographic pictures (difficult explicit), 1-back with neutral pictures (easy neutral) and 2-back with neutral pictures (difficult neutral)). In addition, six regressors of no interest containing the motion parameters were included. Each boxcar stimulus function was convolved with a canonical hemodynamic response function. Then, the data was high pass filtered with a cut-off period of 128 s. At a group level, the contrast images of each subject representing the main effects (difficult > easy and explicit > neutral) and interactions (DIFFICULTY X EXPLICITNESS: explicit (easy > difficult) > neutral (easy > difficult)) and GROUP X EXPLICITNESS: patient (explicit > neutral) > control (explicit > neutral)) were used for a random effect analysis. Next, a two-sided *t*-test was used to assess group differences. The threshold for all analyses was set to p ≤ 0.05 family wise error (FWE) corrected for multiple comparisons on the cluster level. The peak voxel of significant clusters was localized using automatic anatomical labelling (AAL) (Tzourio-Mazoyer et al., 2002).

### 2.12 Psychophysiological interaction

To further explore the mechanisms of how the lingual gyrus region is modulated during processing of pornographic pictures, a psychophysiological interaction (PPI) analysis (Friston et al., 1997) was performed. A PPI analysis reveals differences in functional connectivity between a particular seed region and all other voxels across the entire brain as a function of a psychological factor. Here, we conducted a PPI analysis to identify brain regions that showed differential connectivity between the two groups during processing of pornographic background pictures. We used parts of the left lingual gyrus during pornographic stimulation as the seed, because it showed a SEXUAL BEHAVIOR X EXPLICITNESS interaction of neuronal activity (seed region (x, y, z) (-2, 82, 2)), as identified by the interaction contrast (patients (pornographic > neutral) > controls (pornographic > neutral)) (see Table 3). The blood oxygenation level-dependent time series was extracted from a sphere located in the lingual gyrus (5 mm diameter and centred on the peak voxel) for every subject individually using the first eigen-time series (principal component analysis). The PPI regressor was calculated for each subject as the element-by-element product of the mean-corrected activation of the seed region (extracted time series) and the vector coding for the psychological variable (1 on pornographic regressors and −1 on regressor of the control condition coding for areas affected by processing of pornographic pictures). Thus, our PPI tested for a pornographic-specific modulation of the functional connectivity between the left lingual gyrus and any other brain region. Finally, the individual contrasts reflecting the interaction between the psychological and physiological variables (PPI regressor) were entered into a two-sample *t*-test.

## 3 Results

### 3.1 Demographic

The analysed groups were matched with respect to age (controls 37.6 ± 11.7, patients 36.3 ± 11.2, T(67) = 0.46, p = n.s.), years of education and handedness (four left-handed per group) and did not differ with respect to working memory capacity as indicated by the WAIS-IV Arithmetic subtest (controls: 11.16 ± 2.66 scaled score, patients: 11.16 ± 2.59 scaled score, T(67) = 0.005, p = n.s). For further details, see Table 1.

**Table 1:** **Clinical characteristics**: Mean (M) and standard derivation (SD) of the clinical description of the sample as well as the T-value and the corresponding p-value for the group comparison.

	Patients (M ± SD)	Controls (M ± SD)	T value/p-value
Age	36.3 ± 11.2	37.6 ± 11.7	0.46 / 0.647
Years in school	11.7 ± 1.6	12 ± 1.5	0.849 / 0.399
WAIS IV – arithmetic subtest	107.7 ± 16.6	106.87 ± 15.3	0.22 / 0.826
HBI	73.1 ± 10.9	28.1 ± 8.7	18.624 / >0.001
SAST - R	13.3 ± 3.2	2.1 ± 2.2	16.44 / >0.001
Pornography consumption – last week (min)	213 ± 242	49 ± 70	3.646 / 0.001
Number of orgasms – masturbation (week)	13.1 ± 18.3	2.0 ± 2.5	3.34 / 0.001
SIS-1	35.6 ± 8.2	31.9 ± 5.4	2.274 / 0.026
SIS-2	25.8 ± 5.3	29.8 ± 4.4	3.359 / 0.001
SES	60.5 ± 10.5	49.4 ± 8.5	4.735 / >0.001

### 3.2 Behavioural

To test for group differences in general, working memory performance and reaction times in the neutral conditions were compared between groups. The raw data is presented in Table 2. Here, a 2 × 2 repeated measure analysis with the between subject factor SEXUAL BEHAVIOUR and the within subject factor DIFFICULTY revealed an effect of DIFFICULTY (F(1,67) = 63.318, p < 0.001, η^2^ = 0.486) but no group differences (F(1,67) = 3.604, p = n.s.) for accuracy and again an effect of DIFFICULTY (F(1,67) = 40.471, p < 0.001, η^2^ = 0.377) but no group differences (F(1,67) = 0.317, p = n.s.) for median reaction times.

**Table 2:** **Behavioral performance**: Behavioral data from the n-back task and the surprise recognition task. Depicted are mean (M) and standard derivation (SD) of the two groups as well as the t values of the group comparison (T-value and corresponding p-value).

	Patients (M ± SD)	Controls (M ± SD)	T value/p-value
Accuracy explicit 1-back	93.4% ± 11.1	97.7% ± 4.7	2.136/**0.037**
Accuracy explicit 2-back	80.1% ± 18.6	88.2% ± 10.3	2.274/***0.027***
Accuracy neutral 1-back	95.9% ± 5.9	98.0% ± 3.9	1.788/0.078
Accuracy neutral 2-back	82.3% ± 14.7	87.6% ± 11.9	1.627/0.109
RT explicit 1-back	668 ms ± 113	607 ms ± 75	2.552/**0.013**
RT explicit 2-back	727 ms ± 125	696 ms ± 97	1.149/0.255
RT neutral 1-back	609 ms ± 90	597 ms ± 81	0.57/0.57
RT neutral 2-back	693 ms ± 116	714 ms ± 112	0.765/0.447
Correctly remembered explicit 1-back	65.5% ± 21.0	48.3% ± 21.7	3.299/**0.002**
Correctly remembered explicit 2-back	52.0% ± 19.4	40.0% ± 18.6	2.641/**0.01**
Correctly remembered neutral 1-back	40.0% ± 18.4	46.2% ± 20.3	1.311/0.194
Correctly remembered neutral 2-back	25.3 ± 18.0	34.7% ± 22.0	1.936/0.057

To evaluate effects of pornographic material on working memory, performance data was analysed with a 2 × 2 × 2 repeated measure ANOVA comprising the factors SEXUAL BEHAVIOR (patients/control), EXPLICITNESS (pornographic/neutral) and DIFFICULTY (1-back/2-back).

**Fig. 1:**
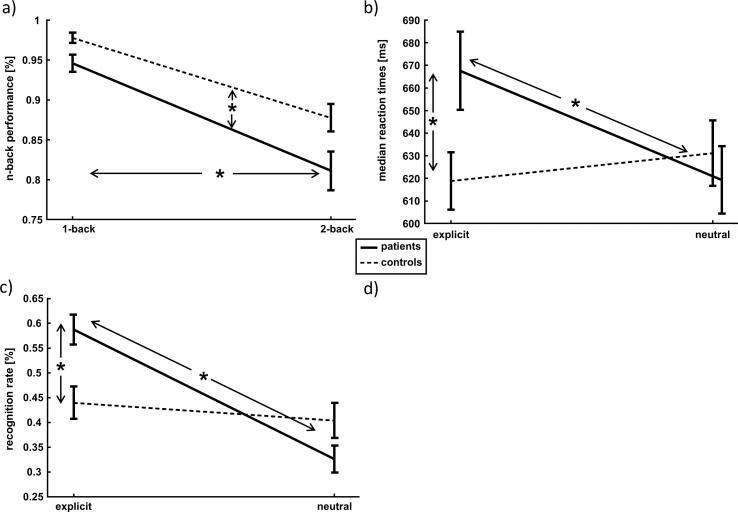
**Behavioral results:** a) Main effect of difficulty and sexual behavior on the accuracy in the n-back task. Subjects perform worse in the more difficult 2-back condition and controls outperform patients independent of the difficulty. Error bars denotes standard error of the mean (SEM). b) Depicted is the sexual behavior X explicitness interaction on median reaction times showing that patients react slower with distracting pornographic material while no differences with neutral images are detected. Error bars denotes standard error of the mean (SEM). c) Sexual behavior X explicitness interaction for the surprise recognition task. Patients show better memory performance for irrelevant pornographic background pictures while no differences for neutral images could be detected. Error bars denotes standard error of the mean (SEM).

Analysis of accuracy revealed a main effect of DIFFICULTY (F(1,67) = 140.758, p < 0.001, η^2^ = 0.678) and SEXUAL BEHAVIOUR (F(1,67) = 5.213, p = 0.026, η^2^ = 0.072) but neither an effect of EXPLICITNESS (F(1,67) = 0.305, p = n.s.) nor an interaction between the factors (see Fig. 1a).

Regarding the median reaction times, rm-ANOVA showed an interaction between SEXUAL BEHAVIOUR and EXPLICITNESS (F(1,67) = 11.73, p = 0.001, η^2^ = 0.149) as well as main effects of DIFFICULTY (F(1,67) = 45.106, p < 0.001, η^2^ = 0.402) and EXPLICITNESS (F(1,67) = 4.142, p = 0.046, η^2^ = 0.058), but neither a main effect of SEXUAL BEHAVIOUR (F(1,67) = 0.868, p = n.s) nor any other significant interaction could be found. Post-hoc t-tests showed that patients reacted slower with sexually explicit distracting pictures compared to healthy controls (T(67) = 2.271, p = 0.027), but both groups performed similarly with neutral stimuli in the background (T(67) = 0.563, p = n.s). In addition, patients reacted slower with explicit compared to neutral stimuli in the background (T(37) = 3.195, p = 0.003), while in healthy controls, only a trend toward significance could be detected (T(30) = 1.956, p = 0.060), which points towards faster reaction times in the explicit conditions (see also Fig. 1b).

**Fig. 2:**
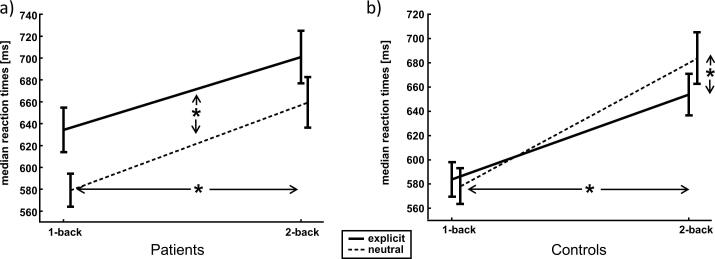
**Behavioral results for the different groups:** a) Main effect of explicitness: Patients react slower with pornographic background pictures independent of the task difficulty. b) Explicitness X difficulty interaction. Healthy controls react faster with pornographic background pictures only in the difficult condition.

For a more detailed look at the distracting effect, we analysed the median reaction times in each group individually. Therefore, a 2 × 2 repeated measure analysis was conducted comprising the factors EXPLICITNESS and DIFFICULTY. In the patient group, we found main effects of EXPLICITNESS (F(1,37) = 10.209, p = 0.002, η^2^ = 0.216) and DIFFICULTY (F(1,37) = 23.021, p < 0.001, η^2^ = 0.384) with faster reaction times in the easy condition and longer reaction times with distracting pornographic pictures, but no interaction between both (see also Fig. 2a). For the control group, on the other hand, a main effect of DIFFICULTY (F(1,30) = 21.736, p < 0.001, η^2^ = 0.42) and a DIFFICULTY × EXPLICITNESS interaction (F(1,30) = 4.606, p = 0.04, η^2^ = 0.133) was detected, but no main effect of EXPLICITNESS (F(1,30) = 3.826, p = n.s.) could be found (see also Fig. 2b). Post hoc t-tests showed that healthy subjects were faster in the more difficult 2-back condition when pornographic pictures were presented (T(30) = 2.666, p = 0.012), while in the easier 1-back condition, response speed was comparable between neutral and pornographic background pictures (T(30) = 0.583, p = n.s.).

In the recognition task, the 2 × 2 × 2 rm-ANOVA revealed a main effect of EXPLICITNESS (F(1,66) = 31.574, p < 0.001, η^2^ = 0.324) and DIFFICULTY (F(1,66) = 85.492, p < 0.001, η^2^ = 0.564) as well as an EXPLICITNESS × SEXUAL BEHAVIOR interaction (F(1,66) = 16.651, p < 0.001, η^2^ = 0.201) for task accuracy. Post hoc t-tests showed a similar memory performance between groups for neutral pictures (T(66) = 1.51, p = n.s.), but a better performance for pornographic material in the patient group (T(66) = 3.097, p = 0 0.003). In addition, the control group performed similarly in neutral and sexually explicit conditions (T(29) = 1.012, p = n.s.), while patients showed a better memory performance for pornographic pictures (T(37) = 7.398, p < 0.001) (see Fig. 1c).

### 3.3 fMRI

**Fig. 3:**
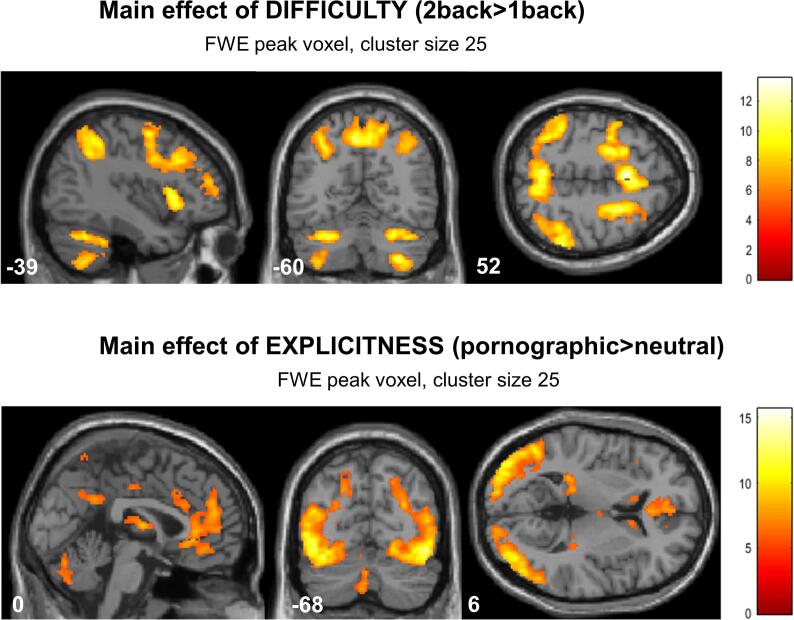
**fMRI main results**: Depicted are the main effects of difficulty, showing higher activation in the fronto-parietal attention network for the more difficult 2back condition as well as the main effect of explicitness showing higher activation in occipital areas as well as anterior cingulate cortex during observation of pornographic pictures.

Sexually explicit pornographic pictures in the background activated large clusters in the occipital cortex and cingulate cortex (anterior, middle and posterior) bilaterally. In addition, a higher activation in the hippocampus and caudate nucleus was observed. In contrast, neutral background pictures led to higher activity in the parahippocampal and angular gyrus. The 2-back task resulted in a higher activation in inferior parietal and inferior frontal areas compared to the 1-back condition (see also Fig. 3 and Table 3).

**Fig. 4:**
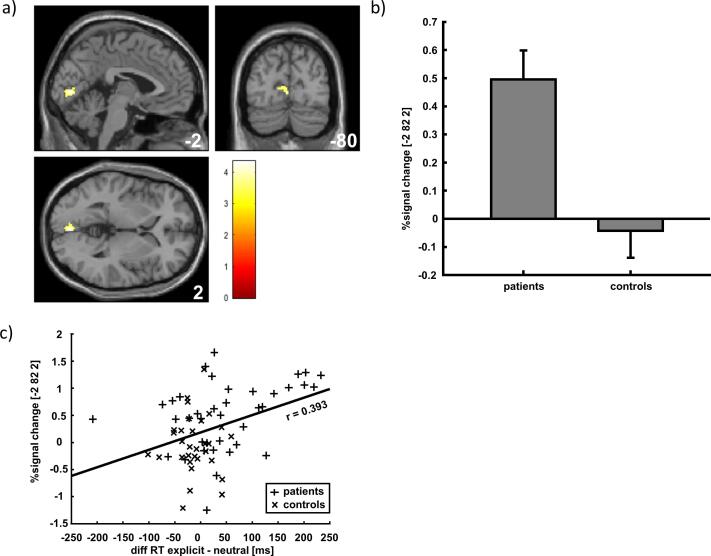
**fMRI interaction result:** A) Shown is the higher activation in the lingual gyrus for patients during presentation of pornographic pictures compared to neutral pictures. B) Parameter estimates of the interaction effect. C) Correlation between parameter estimates and the difference of reaction time (explicit - neutral).

SEXUAL BEHAVIOR × EXPLICITNESS interaction showed higher activation in the left lingual gyrus for patients when processing pornographic material compared to neutral stimuli (see Table 3 for details). Interestingly, parameter estimates of this cluster were positively correlated to the reaction time difference between explicit and neutral background images (r = 0.393, p = 0.001), the mean time of pornography consumption in the last week (r = 0.315, p = 0.009), the number of orgasms through masturbation using pornographic material (r = 0.323, p = 0.007) and the sexual excitation score (SES) (r = 0.41, p = 0.0004). Furthermore, a correlation between the reaction time differences (explicit-neutral) and time watching pornography during the last week (r = 0.254, p = 0.038) could be detected, meaning that a higher amount of time consuming pornography was associated with higher distraction due to pornographic material (see also Fig. 4 and Table 3).

**Table 3:** **fMRI results**: Results of the fMRI analysis. Shown are the peak activations, cluster size and corresponding AAL labels of the peak activation for the different analyzed contrasts as well as the used correction for multiple comparisons (i.e. FWE correction on peak voxels for main effects and on cluster level for interaction effects).

Location (AAL)	hemisphere	x	y	z	Clustersize	p-value	T –value (peak voxel)
Explicitness:							
Explicit > Neutral; *FWE peak > 25*							
inferior occipital gyrus	L	−44	−76	−6	15,139	0	15.65
Posterior orbital frontal cortex	R	28	32	−14	180	0	7.51
Inferior Parietal cortex	R	30	−48	54	589	0	9.42
Superior medial frontal /ACC	L/R	−4	48	20	1694	0	9.21
Thalamus	L/R	0	−10	10	98	0	8.95
Posterior orbital frontal cortex	L	−30	32	−14	229	0	8.55
Caudate nucleus	R	24	−28	28	84	0	8.41
PCC	L/R	−2	−48	28	348	0	8.17
Hippocampus	R	32	–32	−2	109	0	7.36
Insula	L	−34	24	10	40	0	7.25
Caudate nucleus	L	−18	0	30	43	0	7.23
Middle cingulate cortex	R	20	−16	34	38	0	7.15
Middle cingulate cortex	L	–22	−40	36	29	0	6.86
Middle cingulate cortex	L	−2	−18	40	30	0.001	6.64
Caudate nucleus	L	−12	18	8	39	0.001	6.46
Caudate nucleus	R	8	16	6	34	0.002	6.42
Middle frontal 2	L	−26	40	28	28	0.003	6.3
Precuneus	L/R	0	−58	66	41	0.003	6.23
Explicitness:							
Neutral > Explicit; *FWE peak > 25*							
Parahippocampal gyrus	R	24	−28	−16	20	0.001	6,57
Angular gyrus	R	44	−64	52	5	0.007	6.04
Parahippocampal gyrus	L	−18	−36	−12	1	0.029	5.68
Insula	L	−36	−26	20	1	0.037	5.6
*Difficulty:*							
Difficult > Easy; *FWE peak > 25*							
Cerebellum	L	−28	−56	–32	1089	0	13.52
Supplemental motor area	L/R	−4	16	44	6678	0	13.12
Insula	R	34	22	2	1750	0	12.88
cerebellum	R	34	−52	−30	856	0	11.79
Precuneus	L/R	−6	−60	52	4649	0	11.77
Superior Frontal	R	24	12	60	3733	0	11.6
Cerebellum	R	30	−62	−48	499	0	10.94
Cerebellum	L	−6	−52	−56	65	0	8.61
Anterior Orbitofrontal Cortex	R	22	40	−12	47	0	6.85
Cerebellum	R/L	−2	−44	−16	52	0	6.72
*Difficulty:*							
Easy > Difficult; *FWE peak > 25*							
Middle temporal cortex	R	52	−74	4	4580	0	11.11
Precuneus	R/L	6	−50	24	1463	0	10.76
Hippocampus	L	−24	−18	−16	3316	0	10.25
Inferior orbitofrontal cortex	L	−34	34	−12	107	0	10.13
Rolandic operculum	R	54	−4	10	1262	0	9.41
Supplemental motor area	R/L	2	−16	52	540	0	7.03
Superior frontal cortex	L	−12	38	52	80	0	8.53
Middle temporal pole	R	42	22	−34	341	0	6.86
Olfactory	L/R	−2	26	−12	603	0	8.29
Cerebellum	R	26	−76	−34	25	0	7.86
Inferior orbitofrontal cortex	R	38	34	−12	58	0	7.84
Precentral gyrus	R	46	–22	64	279	0	7.77
Middle temporal cortex	L	−58	6	−18	67	0	7.48
Inferior frontal tri	R	52	36	12	51	0	7.04
Middle temporal pole	L	−46	14	−34	61	0	6.92
Superior temporal	L	−54	−6	6	32	0	6.9
Superior medial frontal	L	−6	52	36	37	0	6.88
Cerebellum	L	−28	−80	−34	49	0.001	6.56
Middle temporal	L	−64	−8	−12	51	0.001	6.53
*Difficulty × Explicitness:*							
Explicit (easy > difficult) > Neutral (easy > difficult); *FWE cluster*							
Inferior occipital	L	−44	−70	−6	1804	0.000	6.58
Insula	L	−30	18	−12	271	0.000	5.78
Middle temporal	L	−58	−18	−10	173	0.000	5.02
Inferior parietal	R	32	−48	54	912	0.000	4.83
Inferior temporal	R	48	−62	−4	296	0.000	4.78
Anterior cingulate cortex	L/R	−2	30	26	758	0.000	4.77
Supramarginal gyrus	L	−60	–32	40	193	0.000	4.74
Precueneus	L	−10	−62	70	1433	0.000	4.69
Superior frontal	L	–22	30	50	156	0.001	4.88
Inferior frontal operculum	L	−46	14	32	585	0.000	4.52
Medial orbitofrontal cortex	L/R	−2	46	−8	99	0.013	4.47
Sexual beahvior *× Explicitness:*							
Patient (explicit > neutral) > Control (explicit > neutral); *FWE cluster*							
Lingual gyrus	L	−2	−82	2	84	0,032	4,34

### 3.4 Psychophysiological interaction

Using a 5 mm sphere around the lingual gyrus peak voxel as the seed for a whole-brain PPI analysis to test for functional connectivity differences induced through processing of pornographic pictures (interaction term: patients (pornographic pictures > neutral pictures) > controls (pornographic pictures > neutral pictures)), we found that this area showed a stronger functional connectivity in patients during distracting pornographic stimuli with regions associated with object processing and attention processing, namely the left superior and inferior parietal cortex as well as the insula (see Table 4 for details).

**Table 4:** **PPI results**: Results of the PPI analysis from a seed in the lingual gyrus between groups. Shown are areas which show a higher functional connectivity in the patient group during processing of irrelevant pornographic pictures FWE corrected for multiple comparisons on the cluster level.

Location (AAL)	hemisphere	x	y	z	Cluster size	p-value	T –value (peak voxel)
*Seed:* Lingual gyrus (-2–82 2); FWE cluster level, patients > controls							
Middle temporal	R	48	−52	4	357	0.000	5.27
Cerebellum	R	28	−50	−50	124	0.005	5.14
Insula	R	40	12	6	84	0.036	4.96
Putamen	R	34	−18	−4	173	0.001	4.7
Insula	L	−36	−2	−4	147	0.002	4.69
Superior parietal	L	−24	−52	58	113	0.008	4.61
Middle occipital	L	−42	−68	16	176	0.001	4.49
Middle frontal	L	−40	36	32	81	0.042	4.37
Inferior parietal	L	−44	−36	36	137	0.003	4.27
Postcentral	R	50	–22	40	126	0.005	4.21
Precentral	R	56	2	38	82	0.04	3.94
Inferior occipital	R	40	−76	−16	178	0.000	3.38

Interestingly, the extracted PPI values for the cluster in the insula (MNI: 40 12 6) correlated with the difference in reaction times for explicit and neutral images (r = 0.289, p = 0.016), showing that the more subjects were slowed due to pornographic material, the stronger the functional connectivity between lingual gyrus and insula. See Table 4 for details.

## 4 Discussion

This study investigated the distracting effect of pornographic material on working memory processes in a sample of subjects displaying CSB. On the behavioural level, patients were slowed down by pornographic material depending on the usage of pornography in the last week. This was accompanied by a higher activation in the lingual gyrus. In addition, the lingual gyrus showed a higher functional connectivity to the insula during processing of pornographic stimuli in the patient group. In contrast, healthy subjects revealed faster responses when confronted with pornographic pictures only with high cognitive load.

On the behavioural level, we found that task difficulty and pornographic pictures slowed down reaction time. However, the group × explicitness interaction showed that patients (but not controls) displayed longer reaction times when confronted with distracting pornographic pictures and thus the effect of pornographic pictures seemed to be driven by the patient group. This was supported by the analysis of the individual groups showing that, in healthy controls, reaction times were even facilitated through pornographic pictures, but only in the difficult condition, while in the patient group, pornographic material independent of the difficulty led to slower reaction times. Thus, our data suggest that pornographic pictures differentially affect patients and controls. Furthermore, healthy controls do not seem to remember pornographic material better than neutral pictures, while patients have a better incidental memorization of pornographic material. Based on these findings, we conclude that pornographic material is not able to automatically attract attention in healthy subjects. As in healthy subjects, we observed an effect only in the difficult condition. For further investigations, task difficulty should be increased. However, subjects with excessive sexual behaviour resulting in a high degree of psychological strain are distracted by pornographic material, as they are slowed down in their response when confronted with task-irrelevant pornographic pictures independent of the task difficulty. The behavioural correlation between pornography consumption and reaction time differences are in line with the results of Pekal et al. (2018), showing that tendencies toward internet pornography disorder are related to a higher attentional bias towards pornographic material, and Sklenarik et al. (2019), showing approach tendencies toward pornographic material are related to pornography consumption. Regarding the group of subjects with excessive sexual behaviour, the ~ 50 ms prolonged reaction time in the explicit condition and the ~ 25% better recognition rate during the unannounced recognition task suggests that the subjects explored the distracting pictures in more detail, which led to a better recall afterwards, even though each picture was presented for 1 s independent of the reaction time. Thus, the mere exposure time did not differ between groups. Interestingly, patients had a rather negative image of sexuality due to their experience, leading to a high psychological strain. As it could be shown that the distracting effect of pain is partly mediated by the subjects’ expectations (Sinke et al., 2016, Sinke et al., 2017), it is possible that the slowing down in pleasure processing could also be mediated by the subjects’ attitudes towards pornography. As we did not access the subjects’ expectations towards pornography, we were not able to analyse this, but further investigations should collect information about the subjects’ attitudes towards sexuality/pornography.

On the neural level, the pornographic pictures were processed as expected, as typical areas for processing of visual sexual stimuli were activated, such as the inferior occipital, inferior parietal, orbitofrontal, medial prefrontal, cortex, insula, and anterior cingulate cortex (Stoléru et al., 2012). Furthermore, the more difficult task led to a higher activation in the parietal and frontal areas typically involved in working memory processes (Owens et al., 2018, Takeuchi et al., 2018, Wager and Smith, 2003). The behaviourally relevant observed explicitness × group interaction is mirrored by a differential activation in the lingual gyrus, which is correlated with the distracting effect of the background stimuli. Based on the role of the lingual gyrus for visual encoding (Machielsen et al., 2000), one could speculate that this higher activation reflects the observed better recall for explicit pictures in the patient group. However, we did not find a correlation between recall accuracy and parameter estimates of the lingual gyrus. As the lingual gyrus is also involved in letter processing (Mechelli et al., 2000), it is also possible that the higher activation is caused by a higher effort for patients to focus on the letters. This view is supported through the correlation of the parameter estimates with the reaction time differences between explicit and neutral images, showing that the longer subjects need to react in the explicit condition is, the higher the activation in the lingual gyrus.

Furthermore, we found that the time spent with pornographic material and the orgasms reached through consumption of pornography are correlated with activity in this area, meaning that the more time subjects spend watching pornography and using this material to reach an orgasm, the higher the activation in this area. This could be interpreted in favour of a learning hypothesis in a way that, if someone often consumes pornography (and gets a rewarding orgasm), it is learned that these kinds of stimuli are highly relevant and then the person gets distracted when confronted with related material, similar to the incentive sensitization theory in drug addiction (Robinson and Berridge, 1993, Robinson and Berridge, 2008). This view is supported by a correlation between the reaction time differences and time watching pornography during the last week, showing that the more time spent watching pornography was, the slower the task-related reaction when pornographic stimuli were presented. Interestingly, Gola et al. (2017) found a positive correlation in CSB between pornography consumption and ventral striatal activity during cue processing implying sexual reward which is also in line with the incentive sensitisation theory. In addition Kühn et al. (2014) reported a negative association between gray matter volume of the right caudate nucleus and pornography consumption per week in healthy subjects.

During the processing of pornographic stimuli, the functional connectivity between the lingual gyrus and the network of the middle frontal, superior and inferior parietal, inferior and middle occipital cortex and the insula increases. The insula might especially be an interesting node, as it is a key hub of the salience network (Menon and Uddin, 2010). This could be interpreted in a way that pornographic material has (probably due to learning processes) a high relevance for patients and thus activates the salience (insula) and attention network (inferior parietal), which then leads to a slower reaction time as the salient information is not relevant for the task. Based on these findings, one may conclude that, for subjects displaying CSB, pornographic material has a higher distracting effect and thus a higher salience. Subsequently, the data supports the IST of addiction in CSB.

However we have to note that the study only investigates male heterosexual subjects and that inclusion criteria were defined according to Kafka’s criteria which do not directly translate to ICD-11 criteria.

All in all, we have to conclude that, in healthy subjects, working memory processes are not interrupted by pornographic material and could even be seen as beneficial in demanding tasks. On the other hand, subjects with excessive sexual behaviour are distracted, which is mediated by the lingual gyrus and might be caused by their internal prioritization of sexual stimuli (possibly learned through the excessive coupling of orgasm and pornography consumption) and their negative attitudes towards their sexual behaviour.

## 5 Data and code availability statement

The raw data is available on request from the corresponding author.

## CRediT authorship contribution statement

**C. Sinke:** Conceptualization, Data aquisition, Formal analysis, Visualization, Writing - original draft. **J. Engel:** Project administration, Data acquisition, Writing - reveiw & editing. **M. Veit:** Project administration, Data acquisition, Writing - reveiw & editing. **U. Hartmann:** Conzeptualization, Fund acquisition, Supervision, Writing - reveiw & editing. **T. Hillemacher:** Conzeptualization, Fund acquisition, Writing - reveiw & editing. **J. Kneer:** Conzeptualization, Investigation, Writing - reveiw & editing, Resources. **T.H.C. Kruger:** Conceptualization, Funding acqusition, Project administration, Supervision, Writing - reveiw & editing.

## Declaration of Competing Interest

The authors declare that they have no known competing financial interests or personal relationships that could have appeared to influence the work reported in this paper.
